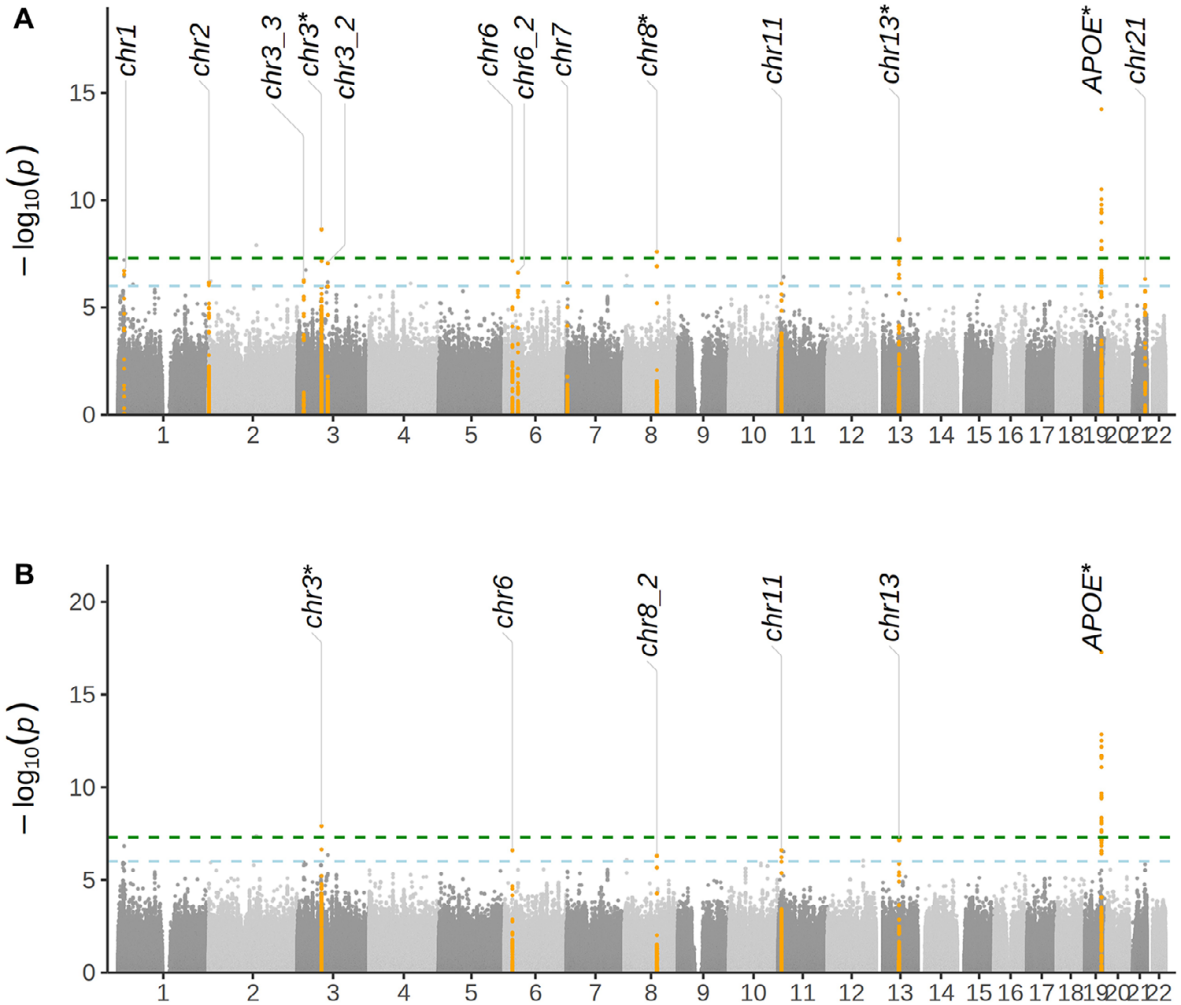# Genome‐wide association study of ADAS scores identifies novel loci linked to cognitive function in Alzheimer’s disease

**DOI:** 10.1002/alz70861_108684

**Published:** 2025-12-23

**Authors:** Mengyuan Kan, Elizabeth Mamourian, Jingxuan Bao, Tianhua Zhai, Saima Rathore, Basavaraj Hooli, Li Shen

**Affiliations:** ^1^ University of Pennsylvania, Philadelphia, PA USA; ^2^ Emory University, Atlanta, GA USA; ^3^ Eli Lilly and Company, Indianapolis, IN USA

## Abstract

**Background:**

Alzheimer’s disease (AD) is characterized by progressive cognitive decline and genetic influences on cognition are well‐established. Investigating single nucleotide polymorphisms (SNPs) associated with cognitive function across dementia progression stages may identify early genetic markers of cognitive decline. The Alzheimer's Disease Neuroimaging Initiative (ADNI), which categorized participants as cognitively normal, mild cognitive impairment, or dementia, provide a valuable resource for such studies. We therefore conducted genome‐wide association study (GWAS) on ADAS scores in ADNI participants, to identify SNPs associated with cognitive performance, serving as early markers of dementia progression.

**Method:**

Genotyped data from three ADNI phases were downloaded and merged. Quality control retained common SNPs with a variant call rate >0.98, sample call rate >0.95, Hardy‐Weinberg Equilibrium *p* ‐value >10^‐6^, and minor allele frequency (MAF) ≥0.01. Individuals were included if their genetic sex matched reported sex and they had no up‐to‐third‐degree relationships with other participants. Genotype imputation was performed using the Michigan Imputation Server. Functional annotation was performed using ANNOVAR. Separate GWAS of ADAS11 and ADAS13 were performed using linear regression in PLINK2, adjusting for age, sex, years of education, and the first ten principal components. Genetic loci were determined based on index SNPs (*p* ‐value <10^‐6^) with more than one nominally associated SNPs (*p* ‐value <10^‐4^) in linkage disequilibrium (r^2^ ≥0.02) within 250kb using PLINK clump function.

**Result:**

A total of 1,236 participants and 8,416,387 common autosomal SNPs were included in the analyses. Thirteen genetic loci were identified as associated with ADAS11, and six with ADAS13, five of which were shared (Figure 1A and 1B). Four index SNPs reached the genome‐wide significant threshold (*p* ‐value <5×10^‐8^), including the strongest signal at the *APOE* locus, primarily driven by dementia, and three novel but less frequent SNPs (MAF <0.05) located on chromosomes 3, 8 and 13. The chromosome 3 locus is near the *SUCLG2* gene, previously reported having an SNP linked to cerebrospinal fluid Aβ_1–42_ levels in AD patients.

**Conclusion:**

Novel genetic loci identified in ADNI provide insights into the genetic basis of cognitive performance, warranting further research on whether well‐established A/T/N imaging or fluid biomarkers mediate their effects on cognitive and diagnostic outcomes.